# Assessment of practice and barriers of oxygen therapy in critically ill patients among nurses: A survey from University of Gondar Comprehensive Specialized Hospital Northwest, Ethiopia, 2021

**DOI:** 10.1016/j.amsu.2022.103481

**Published:** 2022-03-14

**Authors:** Yayeh Adamu Getahun, Yosef Belay Bizuneh, Debas Yaregal Melesse, Wubie Birlie Chekol

**Affiliations:** a Department of Anesthesia, College of Medicine and Health Sciences, Dilla University, Ethiopia; b Department of Anesthesia, College of Medicine and Health Sciences, University of Gondar, Ethiopia

**Keywords:** Practice, Oxygen therapy, Critical illness, Critical care, Nurse, Intensive-care unit

## Abstract

**Background:**

Administering oxygen therapy plays an essential role in preventing and managing acute and chronic hypoxemia. This study assesses the level of practice of nurses on oxygen therapy in critically ill patients and associated factors.

**Methods:**

An institutional-based cross-sectional study was conducted from May 23 to June 07, 2021, at the University of Gondar Comprehensive Specialized Hospital, Northwest Ethiopia. A self-administered structured and validated questionnaire was used. It has a socio-demographic characteristics, multiple choice questions, items that measure the possible associated factors and items were used to assess level of knowledge. Data were entered using Epi-data version 4.6 and analyzed using SPSS version 20. Descriptive and inferential statistics were analyzed and presented. The Chi-Square test was used to measure the strength of associations between variables. Binary and multiple logistic regression were used, a p-value of< 0.2 and < 0.05 were considered statistically significant, respectively.

**Results:**

A total of 400 nurses participated in the study, with a response rate of 94.8%. The overall proportion of good practice on oxygen therapy for critically ill patients was 47% (95% CI: 43–51.8). Age >39 years (AOR; 3.17, 95% CI: 1.42–7.08), nurses have good knowledge on oxygen therapy (AOR; 1.74, 95% CI: 1.11–2.74), labeling of the volume of the cylinder after use (AOR; 2.51, 95% CI: 1.36–4.63), were significantly associated with good practice on oxygen therapy in critically ill patients.

**Conclusions and recommendations:**

We concluded that there was a gap in the practice of oxygen therapy among nurses. Therefore, regular educational and training programs about oxygen therapy are needed to increase the level of practice among nurses. In addition practical training sessions should be organized for nurses to update their practice on the latest guidelines on oxygen therapy for critical ill patients.

## Highlights

• To assess the level of practice of nurses on oxygen therapy in critically ill patients.
• An institutional-based cross-sectional survey study was conducted.
• The level of good practice on oxygen therapy was 47% (95% CI: 43–51.8).
• There is gap in practice of oxygen therapy among nurses.

## 1 Introduction

Critically ill patients obtain supplemental oxygen to prevent or treat hypoxemia [1,2]. Unable to administer oxygen correctly places the patient at risk of hypoxemia, respiratory abnormality, and death [3]. Contrariwise administering too much oxygen also be dangerous, through mechanisms, such as hypoxic pulmonary vasoconstriction, a decrease in tissue oxygen delivery, absorption atelectasis, and the generation of oxygen free radicals [[4], [5], [6], [7]]. From these unfavorable effects are hypoventilation, atelectasis, pulmonary oxygen toxicity, irritation and infection [8,9]. Oxygen therapy is listed as the main item on the World Health Organization model of essential medicines, which is a list of the most effective and safe drugs used in a health care system [10]. Different systems are used for oxygen therapy such as face mask, oxygen tent, nasal cannula, venturi-mask, partial re-breather mask, non-re-breather mask, oxygen hood, face tent, *trans*-tracheal catheter, and nasal catheter [10,11].

Thus, ensuring that supplemental oxygen is administered in a timely and appropriate way is underlying to patient care and the role of a critical care nurse [12]. Nurses regularly and independently manage oxygen therapy in the care of critically ill patients to enhance oxygen delivery and prevent the negative effects of hypoxemia [13]. Oxygen therapy settlements for critically ill patients are, complex because of acute illness, chronic pathology, or perioperative care and are often unsupported by strong evidence [[14], [15], [16], [17]].

Previous studies have identified unpredictability in the oxygen administration practices of emergency and critical care nurses, this unpredictability of oxygen therapy practices of critical care nurses is controversial, given that nurses’ decisions and oxygen therapy management may impress patient outcomes [[18], [19], [20], [21]]. Nurses should have to be skilled in the best practices to keep away from several practical gaps that lead to substandard outcomes for patients like pulse oximetry, humidification attachment, use of different oxygen devices to save the life of many emergency patients [22].

The main factors which were associated with the substandard practice were lack of oxygen therapy training, workload, inadequate supply of oxygen and delivery devices, qualifications, and lack of local guidelines [[23], [24], [25]]. University of Gondar Comprehensive Specialized Hospital is a large hospital with a high flow of critical cases for surgical and medical care which may need the support of oxygen therapy and knowledge of nurses’ practice on oxygen therapy in the hospital is lacking. This study aims to assess the level of practice of nurses toward oxygen therapy in critically ill patients and associated factors.

## 2 Methods

### 2.1 Design, period, and study area

An institution-based cross-sectional study was conducted from May 23 to June 07, 2021, at the University of Gondar Comprehensive Specialized Hospital. The University of Gondar Comprehensive Specialized Hospital is located in Gondar City, Northwest Ethiopia. A statistics report in 2019, showed that the hospital had 450 nurses working in different medical and surgical wards who work in rotation every two years. The Research Registry number was stated as 7557 and, in accordance with the Declaration of Helsinki, 2013. This study has been reported in line with the STROCSS criteria [26].

### 2.2 Variables of the study

Dependent Variable; level of good practice. Independent Variables; Socio demographic factors (level of education, experience, age, sex, and marital status), workload, and working area, and training, availability of local guideline, adequate supply of oxygen and delivery system, payment for oxygen therapy, labeling volume of oxygen in the cylinder.

### 2.3 Sample size determination and sampling technique

#### 2.3.1 Sample size determination

To determine the sample size, a single population proportion formula was used. Since there was a previous study done at Addis Ababa [23] similar to the objective of this topic we take the proportion of 53.3% by assuming a 95% of confidence interval with a 5% margin of error, and finally the sample size for the study is calculated as:

n=(z∝2)2pqε2

Where;

n = is the desired sample size; z = is standard normal distribution usually set as 1.96 (corresponds to 95% confidence level); p = population proportion (53.3%, 0.533), and q which is 1–0.533 = 0.467.
d = degree of accuracy desired (marginal error is 5% (0.05)); then the sample size was
n = $\frac{{\left(1.96\right)}^{2}\times \left(.533\times .467\right)\;}{{\left(0.05\right)}^{2}}$ = 382.486 ≈ 383 but we added a non-response rate of 10% which was 38.3 ≈ 39 so our sample size was 422 which was near equal to the total number of nurses so we took all nurses as a sample size.

#### 2.3.2 Sampling technique

Based on inclusion and exclusion criteria, and institutional cross-sectional survey was conducted.

**Inclusion and Exclusion:** In this study, nurses, who were available at the workplace, were included in the study, and nurses who were not directly involved in bedside patient care such as nurse managers were excluded from the study.

### 2.4 Data collection procedure

A self-administered structured and validated questionnaire was used; the questionnaire was validated by a panel of five nurses. It has socio-demographic characteristics, 11 items multiple-choice questions and finally dichotomized to correct and incorrect, seven items that measure the possible associated factors (Yes, I don't know, no.). Knowledge of nurses was also assessed based on 6 items. Internal consistency among the questionnaire items was 0.90 Cronbach's alpha (α) and it was considered within the acceptable range [27]. The small meeting with unit managers was organized to clarify the idea and procedures of the study and obtain their consent to carry it out. A brief preamble to the participants has individually been organized to obtain the consent forms provided by the researcher to eligible participants at work. The data collector allowed nurses sufficient time to read the consent form and ask questions if any. Each participant has had time to complete the questionnaire in front of the data collector.

### 2.5 Data quality management

Data collectors were provided training by the principal investigator. The data collectors were closely monitored by the principal investigator throughout the study period. Study participants were provided adequate information regarding assessment tools. The collected data were checked for completeness, accuracy, and clarity on the day of data collection before being entered into the database by the principal investigator.

### 2.6 Data processing and analysis

Epi data version 4.6 was used to enter data into the computer, which was then transferred to the Statistical Package for Social Sciences (SPSS) version 20 for analysis. Descriptive and inferential statistics were analyzed and presented. Initially, binary logistic regression was carried out to see the association of each independent variable with the outcome variable. Then after to see the relationship between practice and associated factors, multiple logistic regression was used. The Chi-square test was used to measure the strength of associations between variables. A p-value of <0.05 was considered as statistically significant.

## 3 Results

### 3.1 Socio-demographic characteristics of nurses

A total of 400 nurses participated in the study, with a response rate of 94.8%. Among the study participants, 50.7% were males. The mean age of the study participant was 32.18 ± 5.16 years and 68.5% of study participants were married. Most of the study participants (80.8%) from the total study population have a bachelor degree in nursing. The majority of the study participants (39%) have 4–6 years of working experience in the study area (Table 1).

**Table 1:** Socio-demographic characteristics of nurses at University of Gondar Compressive Specialized Hospital, 2021, (n = 400).

Socio-demographic variables	Classification	Frequency	Percent
Age	20–29	158	39.5
	30–39	207	51.8
	≥40	35	8.8
Gender	Female	197	49.3
	Male	203	50.8
Marital status	Single	123	30.8
	Married	274	68.5
	Divorced	3	0.8
Level of education	Diploma	49	12.3
	Bachelor	323	80.8
	Masters	28	7.0
Year of experience	<1 years	7	1.8
	1–3 years	39	9.8
	4–6 years	156	39
	7–9 years	145	36.3
	≥10 years	53	13.3

### 3.2 Practice of nurses towards oxygen therapy

The mean ± standard deviation of practice score of nurses on oxygen therapy was 3.58 ± 2.27. Based on the practice of supplemental oxygen administration, only 47% (95% CI: 42.3–52.2) of the nurses had a good practice and the majority, 53% (95% CI: 47.8–57.8) of nurses had poor practices of supplemental oxygen administration. Only 59.3% (95% CI: 54.8–64.3) of nurses assessed factors affecting pulse-oximetry reading during supplemental oxygen administration (Table 2).

**Table 2:** Responses to practice questions by nurses; University of Gondar Comprehensive Specialized Hospital, 2021; (N = 400).

Variables	Categories	F(N)	P (%)
Before oxygen administration nurse	Correct	294	73.5
	Incorrect	106	26.5
Special monitoring of the patient on oxygen nurse	Correct	59	14.8
	Incorrect	341	85.3
Pulse oximetry monitoring is affected by	Correct	237	14.8
	Incorrect	163	85.3
The best practice on pulse oximetry	Correct	167	41.8
	Incorrect	233	58.3
Reduce the risk of side effects associated with dry gas administration and to promote patient comfort	Correct	185	46.3
	Incorrect	215	53.8
Collection of water in the tubing during oxygen administration	Correct	66	16.5
	Incorrect	334	83.5
Oxygen cannot travel easily through wet secretions, so optimize their removal by	Correct	141	35.5
	Incorrect	259	64.8
Nasal cannulae	Correct	115	28.8
	Incorrect	285	71.3
Your patient may have difficulty of tolerating and constantly struggling to remove the oxygen delivery device	Correct	128	32
	Incorrect	272	68
High percentage of oxygen 95–100% (Fi02) used for short term treatment	Correct	117	29.2
	Incorrect	283	70.8
Which nursing care is appropriate during oxygen therapy	Correct	191	47.8
	Incorrect	209	52.2

Key; P = percentage; N=Number; F=Frequency.

### 3.3 Factors associated with the practice of nurses towards oxygen therapy

Both binary and multiple logistic regression analyses were done to see the effect of selected characteristics on the practice of nurses. Age, labeling volume of a cylinder and having good knowledge about oxygen therapy were significantly associated with good practice in the multivariate analysis.

The likelihood of nurses having good practice towards oxygen therapy for critically ill patients was 3.17 times higher, among age >39 years (AOR: 3.17, 95% CI: 1.42–7.08) as compared with the age group 20–29 years. The odds of having good practice towards oxygen therapy were 2.51 times higher among nurses who got a volume of cylinder equivalent to the labeled one (AOR: 2.51, 95% CI: 1.36–4.63) as compared with nurses with their counterparts. Finally, nurses, who had good knowledge about oxygen therapy (AOR: 1.74, 95% CI: 1.11–2.74) were 1.74 times more likely to have good practice as compared with nurses who had poor knowledge (Table 3).

**Table 3:** Bivariable and multivariable logistic regression analysis of factors associated with nurses practice towards oxygen therapy in university of Gondar Comprehensive Specialized Hospital, 2021; (n = 400).

Variables	Category Practice		COR (95%CI)	AOR (95%CI)	P
	Poor	Good			
**Age**					
20–29	93	65	1	1	
30–39	107	100	1.34 (0.88–2.03)*	1.27 (0.83–1.960)	0.227
≥40	12	23	2.74 (1.27–5.90)*	3.17 (1.42–7.08)	0.005**
**Guideline**					
Yes	68	73	1.47 (0.89–2.41)*	1.06 (0.59–1.89)	0.83
I don't know	77	66	1.17 (0.72–1.92)*	1.01 (0.59–1.73)	0.985
No	67	49	1	1	
Work load					
Yes	131	131	1.33 (0.77–2.32)*	0.95 (0.51–1.77)	0.864
I don't know	45	30	0.89 (0.45–1.76)*	0.54 (0.25–1.15)	0.111
No	36	27	1	1	
Volume of cylinder labeled					
Yes	37	51	2.12 (1.23–3.67)	2.51 (1.36–4.63)	0.003**
I don't know	95	85	1.34 (0.87–2.17)	1.50 (0.89–2.52)	0.123
No	80	52	1	1	
Knowledge					
Good	56	76	1.89 (1.24–2.88)*	1.74 (1.11–2.74)	0.017**
Poor	156	112	1	1	

**Note:** ** = P < 0.05 and associated in multivariate analysis, * = Factors associated in bivariate analysis; **1** = Constant (Reference); COR = crude odd ratio; AOR = adjusted odds ratio; CI = confidence interval.

## 4 Discussion

Oxygen therapy is the administration of oxygen as a medical intervention which can be used for medical as well as surgical conditions. Patients can be affected by getting too much, too low, or no oxygen, which is mainly determined by the competency and skill of the responsible body administering it. Nurses should know oxygen therapy indications, normal oxygen saturation at different ages, including normal respiration rates [12,[27], [28], [29], [30], [31]].

In this study, based on the practice of supplemental oxygen administration, 47% (95% CI: 42.3–52.2) of the nurses had a good practice. This is in line with research done in Addis Ababa Ethiopia, (43.4%) [23,32], Harari region, Ethiopia (47.5%) [33], and the University Teaching Hospital of Kigali, Rwanda (46.2%) [27]. However, our study was greater than the rates reported by studies conducted in Debra Tabor General Hospital, Ethiopia (33%) [34], southwest, Nigeria (20%) [30] and Cairo, Egypt (40%) [35].

Based on this study, the factors associated with the good practice of oxygen administration were age >39 years, nurses having good knowledge about oxygen therapy and nurses having the habit of labeling the volume of the cylinder after use. This finding is supported by research conducted in Egypt and Addis Ababa, Ethiopia [23,35].

Many years back, several studies have identified that oxygen therapy practices were suboptimal, with oxygen being administered incorrectly at times [36]. They have attributed this to a variety of causes, including failure to administer prescribed treatment [37] and lack of knowledge about the physiological and pharmacological principles of oxygen therapy [19,38]. However, the current practice still indicates that there is a clear practice gap among nurses working on oxygen therapy [33]. Our findings are consistent with those of earlier research about the suboptimal nursing practice of oxygen therapy. Therefore, efforts to improve nurses’ practice of oxygen therapy might result in greater consistency with patient care.

The study of Kord et al. indicated that nurses' age was directly and significantly correlated with the adherence rate to oxygen therapy practice [39]. This was related to an increase in age is directly correlated with increased experience in nurses, nurses’ performance is expected to improve over time as they gain more and more experience [25]. Cousinset al. stated that knowledge of oxygen therapy and the equipment used to deliver oxygen may also be barriers to optimal administration of oxygen [40]. The other study, done in Nigeria, the knowledge of the respondents on oxygen therapy is significantly related to their practices of oxygen administration; implying that those with good knowledge do better in the area of practice [30].

The possible associated leading factors for these findings were identified. A study done in Addis Ababa, in Debre Tabor General Hospital, and Riyadh, showed that lack of oxygen therapy training and guideline, workload, inadequate supply of oxygen and delivery devices were associated factors on practice of oxygen therapy [23,24,34]. Another study showed that the most commonly reported barriers were; the absence of a protocol for oxygen therapy and the unavailability of well-functioning equipment [35]. Unfortunately, these most important factors were not associated with our study.

Participants were also asked about the oxygen therapy guidelines. 141 (35.25%) of nurses response were yes, 143 (35.75%) of nurses response were don't know and 116 (29%) of nurse response were no. In this study, as the majority of nurses (64.75%) evidenced the lack of oxygen therapy guidelines, this could be the start of gaps noted in practice on oxygen therapy (Table 2). The guidelines address the use of oxygen in critically ill and hypoxemic adults, as well as those at risk of hypoxemia [41]. Several guidelines, on oxygen therapy exist including the WHO guidelines and the British Thoracic Society Guideline for oxygen use in adults in healthcare and emergency settings [42,43]. Regarding use of oxygen therapy guidelines, about 1/3 (35.25%) of our study participants were aware of oxygen therapy guidelines which was better than the result of the Nepal survey where only 19.0% of doctors and nurses were aware of the WHO or any other guideline on oxygen therapy [44], however, it was not statistical significant.

## 5 Limitation of the study

The limitation of our study, it was a cross sectional design so it did not show temporal relationships. In addition, our study is a single centered study, which questions the generalizability of our results and all study populations didn't work in the critical care area during the study period.

## 6 Conclusions and recommendations

We concluded that there is gap in practice of oxygen therapy among nurses. Therefore, regular educational and training programs about oxygen therapy are needed to increase the level of practice among nurses. In addition practical training sessions should be organized for nurses to update their practice on the latest guidelines on oxygen therapy for critical ill patients.

## Availability of data

All data generated or analyzed during this study were included in this published article.

## Sources of funding

University of Gondar.

## Author contribution

All authors made a significant contribution to the work reported, whether that is in the conception, study design, execution, acquisition of data, analysis and interpretation, or in all these areas; took part in drafting, revising or critically reviewing the article; gave final approval of the version to be published; have agreed on the journal to which the article has been submitted; and agree to be accountable for all aspects of the work.

## Ethical approval

Ethical clearance to conduct the research was obtained from the ethical review committee of the school of Medicine, College of Medicine and Health Sciences. Written informed consent was obtained from each study participant after a clear explanation of what they would have to do and take part in the study.

## Trial registry number

1. Name of the registry: research registry
Unique Identifying number or registration ID**: 7557**
2. Hyperlink to your specific registration (must be publicly accessible and will be checked): https://www.researchregistry.com/browse-the-registry#home/

## Guarantor

phanuelyosef@gmail.com.

Phone number:+251946460160; p.o.box:196.

## Consent

Not applicable.

## Provenance and peer review

Not commissioned, externally peer-reviewed.

## Declaration of competing interest

There is no conflicts of interest.

## Acronyms and abbreviations

GA: General Anesthesia
COR: Crude Odds Ratio
AOR: Adjusted Odds Ratio
CI: Confidence Interval
ASA: American Society of Anesthesiologists
Hr.: Hour
